# Mysteries of TGF-β Paradox in Benign and Malignant Cells

**DOI:** 10.3389/fonc.2014.00094

**Published:** 2014-05-13

**Authors:** Qiang Zhang, Nengwang Yu, Chung Lee

**Affiliations:** ^1^Department of Urology, Northwestern University School of Medicine, Chicago, IL, USA; ^2^Department of Urology, General Hospital of Jinan Military Command, Jinan, China; ^3^Department of Surgery, NorthShore University HealthSystem, Evanston Hospital, Evanston, IL, USA; ^4^Department of Pathology and Laboratory Medicine, University of California at Irvine, Irvine, CA, USA; ^5^Department of Urology, University of California at Irvine, Irvine, CA, USA

**Keywords:** TGF-β paradox, TGF-β receptors, Erk activation, Smad activation, PP2A recruitment, TGF-β auto-induction, negative feedback, positive feedback

## Abstract

TGF-β regulates a wide range of biological functions including embryonic development, wound healing, organogenesis, immune modulation, and cancer progression. Interestingly, TGF-β is known to inhibit cell growth in benign cells but promote progression in cancer cells; this phenomenon is known as TGF-β paradox. To date, the mechanism of this paradox still remains a scientific mystery. In this review, we present our experience, along with the literature, in an attempt to answer this mystery. First, we observed that, on TGF-β engagement, there is a differential activation of Erk between benign and cancer cells. Since activated Erk is a major mediator in tumor progression and metastasis, a differentially activated Erk represents the answer to this mystery. Second, we identified a key player, PP2A-B56α, which is differentially recruited by the activated type I TGF-β receptor (TBRI) in benign and tumor cells, resulting in differential Erk activation. Finally, TGF-β stimulation leads to suppressed TBRs in tumor cells but not in benign cells. This differentially suppressed TBRs triggers differential recruitment of PP2A-B56α and, thus, differential activation of Erk. The above three events explain the mysteries of TGF-β paradox. Understanding the mechanism of TGF-β paradox will help us to predict indolent from aggressive cancers and develop novel anti-cancer strategies.

## Introduction

TGF-β was initially described in 1982 (1). It was already known to have bi-functional effects, as it can stimulate or inhibit growth of the same cell, depending on conditions (2). These initial reports have demonstrated the mysterious and important nature of TGF-β in physiology and pathology. More than 40 years later, our understanding of TGF-β signaling has greatly expanded and TGF-β is known as an important mediator in cancer progression. In this review, we provide insights into the mystery of the well-known phenomenon of “TGF-β paradox,” mainly based on our own experience, along with the literature information. It should be pointed out that this report is limited to few salient aspects of TGF-β signaling relevant to the present discussion. For a more comprehensive description of TGF-β signaling, please refer to our recent review (3).

## Biology of TGF-β Signaling

There are three known mammalian isoforms of TGF-β (TGF-β1, -β2, and -β3) with significant structural and functional similarity (4). The biological effect of TGF-β is mediated through type I and type II receptors (TBRI and TBRII) (5). The canonical downstream events involve the activation of Smad pathways (6). TGF-β first binds to TBRII, which recruits and activates TBRI (5, 7). The latter then activates Smad2/3. The activated Smad2/3 combines with Smad4 and migrates to the nucleus to regulate transcription (8). In addition to the Smad pathway, TGF-β also signals through a number of non-canonical pathways, including m-TOR, RhoA, Ras, MAPK, PI3K/AKT, PP2A/p70s6K, and JNK (9). The relative importance and interplay of these pathways of TGF-β signaling is still under investigation (10, 11). In this review, we will limit our discussion to TGF-β-mediated Smad and Erk activation.

## TGF-β Paradox

TGF-β is known to inhibit cell cycle in benign cells but promote progression and metastasis in cancer cells (3, 12), a phenomenon known as TGF-β paradox (13). Although there are numerous articles with different approaches tackling this topic, to date, a logical explanation leading to TGF-β paradox remains elusive and is accepted as a scientific mystery (3, 13–15). In this study, we searched the recent literature, along with our own experience, in an attempt to explain this mystery.

## Mystery of TGF-β Paradox 1

### Differential activation of Erk between benign and cancer cells

It is well-known that TGF-β is able to activate Erk in cancer cells (16–18) and inactivate Erk in non-cancer cells (19). However, a direct link of TGF-β-mediated differential activation of Erk between cancer and non-cancer cells in the same cell system has not been reported until our recent report (20), where we treated benign cells with a low concentration of TGF-β (0.1 ηg/ml), which led to Erk activation; while the treatment of the same cells with a high concentration of TGF-β (10 ηg/ml) resulted in Erk inactivation. Activated Erk is a key regulator for cell proliferation. Consistent with this finding, we have observed cell proliferation in benign cells with a low dose of TGF-β but growth arrest with a high dose in benign stromal cells (21) as well as in benign epithelial cells (17). The use of different dosages of TGF-β in these studies is critical as they bring out the interesting phenomenon of differential responses to TGF-β stimulation. It should be pointed out that cancer cells in the early stage of carcinogenesis retain some of the features of benign cells in which they can be inhibited by TGF-β (22, 23). However, in advanced cancer cells, treatment with TGF-β would result in Erk activation and cell proliferation (16, 17, 21, 24).

**Figure 1:**
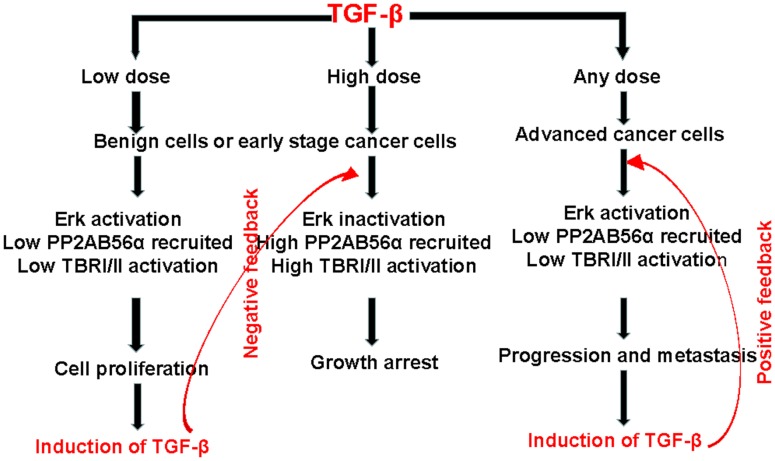
**A proposed mechanism to explain the TGF-β paradox between benign and tumor cells**. First, in benign cells, a low dose of TGF-β in the microenvironment will result in Erk activation and cell proliferation. At the same time, there will be an auto-induction of TGF-β, which will create a high dose of TGF-β in the microenvironment and lead to growth arrest. Therefore, the level of TGF-β is regulated via a negative feedback loop. On the other hand, in cancer cells, Erk will be activated regardless of the level of TGF-β in the microenvironment. The activated Erk is a master regulator of tumor progression and metastasis. It will also auto-induce more TGF-β, which will create a positive feedback of TGF-β signaling in tumor progression. The level of TGF-β is regulated via a positive feedback loop.

The above explanation to TGF-β paradox is summarized in Figure 1. An important point is that, in contrast to the traditional concept of TGF-β paradox (13, 17), TGF-β treatment in benign cells does not always result in growth arrest. Figure 1 indicates that under normal physiological conditions, cellular activities are carefully monitored by TGF-β. Differential Erk activation seems to play a central role in this regulation. When TGF-β level in the local environment is low, cells will activate Erk and induce TGF-β expression (20). On the other hand, when the local concentration of TGF-β is more than sufficient, cells have a mechanism to shut off Erk activation, thus, prevent further expression of TGF-β.

**Figure 2:**
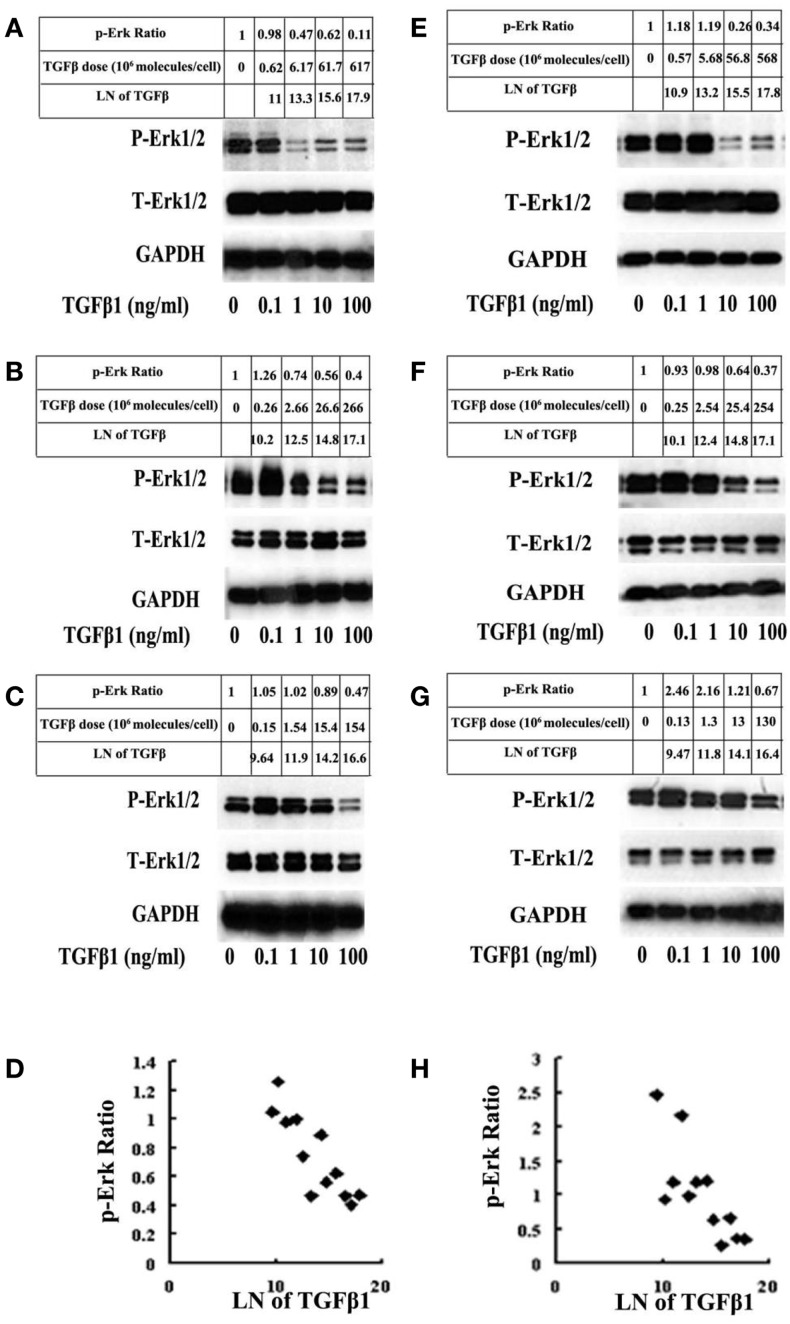
**BPH1 and RWPE1 are immortalized benign prostate epithelial cells**. The cells were treated with 12 different doses of TGF-β1 for 5 min and probed for p-Erk and total Erk (T-Erk) by western blot analysis. A linear relationship between the cellular response (relative value of p-Erk corrected for GAPDH in natural logarithm) and the available level of TGF-β molecules per cell (in natural logarithm) was plotted according to the method described by Clarke et al. (25). In response to a low number of TGF-β1 molecules, there is an increase in the level of Erk. As the number of TGF-β1 molecules increases, there is a linear decline in the relative level of p-Erk in BHP1 cells [**(A–D)**; *r* = −0.86, *p* < 0.05]. A similar phenomenon was observed on RWPE1 cells [**(E–H)**; *r* = −0.77, *p* < 0.05]. There is no change in the amount of T-Erk. Unlike benign cells, malignant cells (DU145 and PC3) always showed an activation of Erk regardless of the dosage of TGF-β1 used in the experiment (data not shown).

It is important to note that Erk activation or inactivation by TGF-β in benign cells is not a case of all-or-none phenomenon. In order to demonstrate the gradual changes in Erk or Smad activation in benign cells, multiple doses of TGF-β at different cell density must be employed as described by Clarke et al. (25). Indeed, they demonstrated a linear increment of Smad activation within a wide range of available TGF-β per cell in mink lung epithelial cells (25). In an attempt to validate the same linear relationship exists between TGF-β dosage and Erk inactivation, we repeated the same experiment performed by Clarke et al. (25) by using a different set of benign epithelial cells (RWPE1 and BPH1). Indeed, a linear Erk inactivation was demonstrated (Figure 2). This phenomenon is applied only to benign cells or early-stage cancer cells, as in advanced cancer cells, there will be no such linear relationship in Smad activation and Erk inactivation upon TGF-β stimulation. In advanced cancer cells, Erk is constantly in an activated state (17, 20) and Smad activation is suppressed, regardless of the level of TGF-β employed. This finding has an important implication in TGF-β paradox, that is, in benign cells or early stage cancer cells, TGF-β offers a mechanism for homeostasis; whereas in advanced cancer cells, it promotes tumor progression (Figure 1).

## Mystery of TGF-β Paradox 2

### Differential recruitment of PP2A-B56α by activated TBRI

The observation of a differentially activated Erk between benign and cancer cells offers an explanation to the mystery of TGF-β paradox (Figure 1). The question remains as how the differentially activated Erk is regulated? Our recent observation has identified a key player, PP2A-B56α (subunit B56α of protein phosphatase 2A), which plays a pivotal role in the regulation of differentially activated Erk between benign and cancer cells upon TGF-β engagement (20). PP2A is a known tumor suppressor (26, 27) and is involved in a broad range of cellular processes, including signal transduction, transcriptional regulation, and control of the cell cycle. The PP2A holoenzyme is a heterotrimer that consists of a core dimer, which has scaffold (A) and catalytic (C) subunits that associate with a variety of regulatory (B) subunits. PP2A-A brings the PP2A-B and -C subunits together. PP2A-C performs the enzymatic reaction, while PP2A-B is responsible to direct the cellular localization and site specificity. The B subunits have been divided into gene families named B (or PR55), B′ (or B56 or PR61), and B″ (or PR72) (28). These B subunits determine the substrate specificity as well as the spatial and temporal functions of PP2A (28). The B56 family consists of B56α, β, γ, δ, and ε, generating at least eight isoforms (28).

TGF-β is known to activate PP2A-Bα in benign cells (26). Only recently, we and others have observed a differential recruitment of PP2A-B56α (but not other PP2As) by the activated TBRI in benign and cancer cells (20, 29). Upon TGF-β treatment in benign cells, TBRII is able to recruit and activate TBRI (5, 7). We found that PP2A-B56α was able to be co-precipitated with the activated TBRI in benign cells in large quantities, leading to inactivation of Erk (20). But in cancer cells, since there is a limited available amount of activated TBRI (30), a limited or no PP2A-B56α was able to be co-precipitated with TBRI, resulting in activation of Erk (20, 31). PP2A is ubiquitously synthesized but is specifically recruited and activated by the activated TBRI to function as an inhibitor to Erk activation (20, 31). This observation was independently validated by others using a different cell system (29).

A key event in TGF-β paradox is the recruitment of PP2A-B56α by the activated TBRI (20, 29). In benign cells, PP2A-B56α is recruited by the activated TBRI, which is dependent on the dosage of TGF-β used to stimulate the target cells. At a high dose of TGF-β, a sufficient quantity of PP2A-B56α is recruited by the activated TBRI, resulting in an inhibition of Erk activation; while at a low dose of TGF-β, due to a limited quantity of TBRI being activated, there will be a limited quantity of PP2A-B56α to be recruited resulting in Erk activation. In the context of advanced cancer cells, due to a severely down-regulated TBRI (30), recruitment of PP2A-B56α is always compromised regardless of the dosage of TGF-β employed, resulting in an elevated activation of Erk (20). This PP2A-B56α-mediated differential Erk activation between benign and malignant cells offers an answer to the mystery of TGF-β paradox.

## Mystery of TGF-β Paradox 3

### Differential suppression of TBRs and auto-induction of TGF-β

Finally, we ask what are the triggering factor and the consequence of the observed differentiated Erk activation in TGF-β paradox. We conclude that the downregulation of TBRs is the triggering factor and the over expression of TGF-β is the consequence of the TGF-β paradox (15). Both events have important implications.

It is well-known that, in advanced cancer cells, TGF-β mediates downregulation of TBRs and auto-induction of TGF-β in cancer cells but not in benign cells (31–34). Since the activated Erk is a master regulator for tumor progression (16), it is responsible for a host of oncogenic signaling events including NF-κB activation, which up-regulates DNA methyltransferases (DNMTs) (35). Targets of DNMTs promoter methylation in many tumor suppressor genes are TBRs (34, 35). A search of the literature has revealed that downregulation of TBRs is an early event of carcinogenesis for all types of cancer (36). The biological consequence of a downregulated TBR will be an attenuate Smad2/3 activation and an elevated Erk1/2 activation in advanced cancer cells. The availability of TBRs dictates the relative levels of activated Erk1/2 and inactivated Smad2/3, thus determines the fate of the TGF-β paradox (25, 37, 38). It follows that any condition that results in downregulation of functional TBRs, such as inflammation (39, 40), Ras activation (41, 42), and loss-of-function mutations in TBRs (43–45), will be predisposed to development and progression of cancer.

TGF-β overproduction is also an universal event in cancer cells and is a poor prognostic marker (20, 35, 46–49). The mechanism, although which TGF-β regulates its own production, is different between benign and cancer cells. Under the normal physiological conditions, the level of TGF-β is tightly regulated within the microenvironment through a negative feedback loop (Figure 1) to maintain a relatively constant level of TGF-β. Too little or too much TGF-β will have an unfavorable consequence (36, 50, 51). However, this principle does not apply to cancer. Cancer cells, especially the advanced cases, are capable of evading the immune surveillance program due to the well-known phenomenon of auto-induction of TGF-β by cancer cells (20), resulting in an elevated TGF-β in the microenvironment through a positive feedback loop (52). As a result, there is an accumulation of TGF-β in the microenvironment, which further promotes tumor progression (20, 35, 48). Therefore, with regard to TGF-β signaling, a characteristic feature of cancer cells, as opposed to the benign cells, is suppressed TBRs (the cause) and an elevated TGF-β (the effect). This feature applies to all cancer cells and can be used as a biomarker for prediction of aggressiveness of the cancer (17, 35).

## Conclusion

### Positive feedback of TGF-β signaling in tumor progression

In summary, this review indicates that a differential Erk activation plays a central role in deciding whether the target cell will undergo growth arrest or proliferation in response to TGF-β. In addition, the description of the conventional concept of “TGF-β Paradox” (12–15) may require a slight modification. First, the term TGF-β paradox does not imply that TGF-β inhibits cell cycle in benign cells. As indicated in this review, benign cells can also be stimulated by TGF-β to undergo proliferation, if the dose of TGF-β is low. Furthermore, cancer cells can be inhibited by TGF-β, especially in the early stages of carcinogenesis, if a sufficient level of TBRs can be activated (Figure 1). Therefore, we conclude that a more appropriate interpretation for TGF-β paradox should be that TGF-β mediates cellular homeostasis in benign cells but promotes tumor progression and metastasis in advanced cancer cells. Second, the term of TGF-β paradox does not imply an all-or-none phenomenon (13, 17). In fact, the changes in target cells in the level of Erk1/2 activation or Smad2/3 inactivation mediated by TGF-β are gradual depending on the relative levels of TGF-β present available per cell in the local microenvironment [Ref. (25), Figure 2]. Also, TGF-β-mediated changes in cell proliferation or growth arrest takes place in a gradual manner depending on the dosage of TGF-β employed (20, 35).

The consequence of Erk activation in cancer cells can result in a continuous TGF-β auto-induction via a positive feedback loop (Figure 1). This continuous production of TGF-β in the tumor microenvironment will further stimulate tumor progression and metastasis resulting in the manifestation of the development of a more aggressive tumor progression. The implications of this positive feedback of TGF-β signaling in tumor progression are at least twofold. First, this knowledge can be used for the prediction of cancer outcome in that TGF-β content in the tumor can be used to predict whether or not the tumor in question is indolent or aggressive (17, 35). Second, due to the knowledge that TGF-β in the tumor microenvironment is highly immune-suppressive (53), it will be important to render the cytotoxic cells insensitive to TGF-β in cancer immunotherapy (54).

## Conflict of Interest Statement

The authors declare that the research was conducted in the absence of any commercial or financial relationships that could be construed as a potential conflict of interest.

## References

[1] Anzano MA Roberts AB Meyers CA Komoriya A Lamb LC Smith JM Synergistic interaction of two classes of transforming growth factors from murine sarcoma cells. Cancer Res (1982) 42:4776–8 6290046

[2] Roberts AB Anzano MA Wakefield LM Roche NS Stern DF Sporn MB Type beta transforming growth factor: a bifunctional regulator of cellular growth. Proc Natl Acad Sci U S A (1985) 82:119–23 10.1073/pnas.82.1.119 3871521 PMC396983

[3] Principe DR Doll JA Bauer J Jung B Munshi HG Bartholin L TGF-β: duality of function between tumor prevention and carcinogenesis. J Natl Cancer Inst (2014) 106:djt369 10.1093/jnci/djt369 24511106 PMC3952197

[4] Patil AS Sable RB Kothari RM An update on transforming growth factor-β (TGF-β): sources, types, functions and clinical applicability for cartilage/bone healing. J Cell Physiol (2011) 226:3094–103 10.1002/jcp.22698 21344394

[5] Massagué J Gomis RR The logic of TGF-β signaling. FEBS Lett (2006) 580:2811–20 10.1016/j.febslet.2006.04.033 16678165

[6] Shi Y Massagué J Mechanisms of TGF-β signaling from cell membrane to the nucleus. Cell (2003) 113:685–700 10.1016/S0092-8674(03)00432-X 12809600

[7] Derynck R Akhurst RJ Balmain A TGF-beta signaling in tumor suppression and cancer progression. Nat Genet (2001) 29:117–29 10.1038/ng1001-117 11586292

[8] Vogelmann R Nguyen-Tat MD Giehl K Adler G Wedlich D Menke A TGF-β induced downregulation of E-cadherin-based cell-cell adhesion depends on PI3-kinase and PTEN. J Cell Sci (2005) 118:4901–12 10.1242/jcs.02594 16219695

[9] Mu Y Gudey SK Landström M Non-Smad signaling pathways. Cell Tissue Res (2012) 347:11–20 10.1007/s00441-011-1201-y 21701805

[10] Miyazono K Transforming growth factor-beta signaling in epithelial-mesenchymal transition and progression of cancer. Proc Jpn Acad Ser B Phys Biol Sci (2009) 85:314–23 10.2183/pjab.85.314 19838011 PMC3621568

[11] Gomes LR Terra LF Wailemann RA Labriola L Sogayar MC TGF-β1 modulates the homeostasis between MMPs and MMP inhibitors through p38 MAPK and ERK1/2 in highly invasive breast cancer cells. BMC Cancer (2012) 19(12):26 10.1186/1471-2407-12-26 22260435 PMC3277461

[12] Inman GJ Switching TGFβ from a tumor suppressor to a tumor promoter. Curr Opin Genet Dev (2011) 21:93–9 10.1016/j.gde.2010.12.004 21251810

[13] Morrison CD Parvani JG Schiemann WP The relevance of the TGF-β paradox to EMT-MET programs. Cancer Lett (2013) 341:30–40 10.1016/j.canlet.2013.02.048 23474494 PMC3752409

[14] Ranganathan P Agrawal A Bhushan R Chavalmane AK Kalathur RK Takahashi T Expression profiling of genes regulated by TGF-beta: differential regulation in normal and tumour cells. BMC Genomics (2007) 8:98 10.1186/1471-2164-8-98 17425807 PMC1858692

[15] Chung SW Cooper CR Farach-Carson MC Ogunnaike BA A control engineering approach to understanding the TGF-â paradox in cancer. J R Soc Interface (2012) 9:1389–97 10.1098/rsif.2011.0799 22188767 PMC3350743

[16] Lee MK Pardoux C Hall MC Lee PS Warburton D Qing J TGF-beta activates Erk MAP kinase signalling through direct phosphorylation of ShcA. EMBO J (2007) 26:3957–67 10.1038/sj.emboj.7601818 17673906 PMC1994119

[17] Zhang Q Helfand BT Jang TL Zhu LJ Chen L Yang XJ NF-kB-mediated transforming growth factor-β-induced expression of vimentin is an independent predictor of biochemical recurrence after radical prostatectomy. Clin Cancer Res (2009) 15:3557–67 10.1158/1078-0432.CCR-08-1656 19447876

[18] Iwanaga R Wang CA Micalizzi DS Harrell JC Jedlicka P Sartorius CA Expression of Six1 in luminal breast cancers predicts poor prognosis and promotes increases in tumor initiating cells by activation of extracellular signal-regulated kinase and transforming growth factor-beta signaling pathways. Breast Cancer Res (2012) 14:R100 10.1186/bcr3219 22765220 PMC3680936

[19] Luo X Zhang Q Liu V Xia Z Pothoven KL Lee C Cutting edge: TGF-beta-induced expression of Foxp3 in T cells is mediated through inactivation of ERK. J Immunol (2008) 180:2757–61 10.4049/jimmunol.180.5.2757 18292494 PMC4289405

[20] Yu N Kozlowski JM Park II Chen L Zhang Q Xu D Over-expression of transforming growth factor β1 in malignant prostate cells is partly caused by a runaway of TGF-β1 auto-induction mediated through a defective recruitment of protein phosphatase 2A by TGF-β type I receptor. Urology (2010) 76: 1519.e8–13 10.1016/j.urology.2010.03.061 21030067 PMC2997920

[21] Zhou W Park I Pins M Kozlowski JM Jovanovic B Zhang J Dual regulation of proliferation and growth arrest in prostatic stromal cells by transforming growth factor-β1. Endocrinology (2003) 144:4280–4 10.1210/en.2003-0554 12959966 PMC1364460

[22] Yamazaki K Masugi Y Sakamoto M Molecular pathogenesis of hepatocellular carcinoma: altering transforming growth factor-â signaling in hepatocarcinogenesis. Dig Dis (2011) 29:284–8 10.1159/000327560 21829019

[23] Kowli S Velidandla R Creek KE Pirisi L TGF-β regulation of gene expression at early and late stages of HPV16-mediated transformation of human keratinocytes. Virology (2013) 447:63–73 10.1016/j.virol.2013.08.034 24210100 PMC3895483

[24] Gore AJ Deitz SL Palam LR Craven KE Korc M Pancreatic cancer-associated retinoblastoma 1 dysfunction enables TGF-β to promote proliferation. J Clin Invest (2014) 124:338–52 10.1172/JCI71526 24334458 PMC3871249

[25] Clarke DC Brown ML Erickson RA Shi Y Liu X Transforming growth factor beta depletion is the primary determinant of Smad signaling kinetics. Mol Cell Biol (2009) 29:2443–55 10.1128/MCB.01443-08 19223462 PMC2668365

[26] Petritsch C Beug H Balmain A Oft M TGF-β inhibits p70 S6 kinase via protein phosphatase 2A to induced G1 arrest. Genes Develop (2000) 14:3093–101 10.1101/gad.854200 11124802 PMC317138

[27] Khanna A Kauko O Böckelman C Laine A Schreck I Partanen JI Chk1 targeting reactivates PP2A tumor suppressor activity in cancer cells. Cancer Res (2013) 73:6757–69 10.1158/0008-5472.CAN-13-1002 24072747 PMC3870284

[28] McCright B Rivers AM Audlin S Virshup DM The B56 family of protein phosphatase 2A (PP2A) regulatory subunits encodes differentiation-induced phosphoproteins that target PP2A to both nucleus and cytoplasm. J Biol Chem (1996) 271:22081–9 10.1074/jbc.271.36.22081 8703017

[29] Samuel GH Bujor AM Nakerakanti SS Hant FN Trojanowska M Autocrine transforming growth factor β signaling regulates extracellular signal-regulated kinase 1/2 phosphorylation via modulation of protein phosphatase 2A expression in scleroderma fibroblasts. Fibrogenesis Tissue Repair (2010) 3:25 10.1186/1755-1536-3-25 21134273 PMC3008687

[30] Zhang Q Rubenstein JN Jang TL Pins M Javonovic B Yang X Insensitivity to transforming growth factor-β signaling is resulted from promoter methylation of cognate receptors in human prostate cancer cells (LNCaP). Mol Endocrinol (2005) 19:2390–9 10.1210/me.2005-0096 15905358

[31] Letourneux C Rocher G Porteu F B56-containing PP2A dephosphorylate ERK and their activity is controlled by the early gene IEX-1 and ERK. EMBO J (2006) 25:727–38 10.1038/sj.emboj.7600980 16456541 PMC1383561

[32] Lee C Sintich SM Mathews EP Shah AH Kundu SD Perry KT Transforming growth factor-β in benign and malignant prostate. Prostate (1999) 39:285–90 10.1002/(SICI)1097-0045(19990601)39:4<285::AID-PROS9>3.0.CO;2-7 10344218

[33] Kim SJ Angel P Lafyatis R Hattori K Kim KY Sporn MB Autoinduction of transforming growth factor beta 1 is mediated by the AP-1 complex. Mol Cell Biol (1990) 10:1492–7 2108318 10.1128/mcb.10.4.1492-1497.1990 PMC362252

[34] Halder SK Cho YJ Datta A Anumanthan G Ham AJ Carbone DP Elucidating the mechanism of regulation of transforming growth factor β type II receptor expression in human lung cancer cell lines. Neoplasia (2011) 13:912–22 10.1593/neo.11576 22028617 PMC3201568

[35] Zhang Q Chen L Helfand BT Zhu LJ Kozlowski J Minn A Transforming growth factor-β-induced DNA methyltransferase contributes to aggressive prostate cancer phenotypes and predicts biochemical recurrence after radical prostatectomy. PLoS One (2011) 6:e25168 10.1371/journal.pone.0025168 21980391 PMC3184137

[36] Singh N Liu G Chakrabarty S Cellular responses to TGFβ and TGFβ receptor expression in human colonic epithelial cells require CaSR expression and function. Cell Calcium (2013) 53:366–71 10.1016/j.ceca.2013.04.003 23639611

[37] Nickl-Jockschat T Arslan F Doerfelt A Bogdahn U Bosserhoff A Hau P An imbalance between Smad and MAPK pathways is responsible for TGF-beta tumor promoting effects in high-grade gliomas. Int J Oncol (2007) 30:499–507 10.3892/ijo.30.2.499 17203233

[38] Briones-Orta MA Tecalco-Cruz AC Sosa-Garrocho M Caligaris C Macías-Silva M Inhibitory Smad7: emerging roles in health and disease. Curr Mol Pharmacol (2011) 4:141–53 10.2174/1874-470211104020141 21222648

[39] Bohrer LR Schwertfeger KL Macrophages promote fibroblast growth factor receptor-driven tumor cell migration and invasion in a CXCR2-dependent manner. Mol Cancer Res (2012) 10:1294–305 10.1158/1541-7786.MCR-12-0275 22893608 PMC3553584

[40] Achyut BR Bader DA Robles AI Wangsa D Harris CC Ried T Inflammation-mediated genetic and epigenetic alterations drive cancer development in the neighboring epithelium upon stromal abrogation of TGF-β signaling. PLoS Genet (2013) 9:e1003251 10.1371/journal.pgen.1003251 23408900 PMC3567148

[41] Ijichi H Chytil A Gorska AE Aakre ME Fujitani Y Fujitani S Aggressive pancreatic ductal adenocarcinoma in mice caused by pancreas-specific blockade of transforming growth factor-beta signaling in cooperation with active Kras expression. Genes Dev (2006) 20:3147–60 10.1101/gad.1475506 17114585 PMC1635149

[42] Trobridge P Knoblaugh S Washington MK Munoz NM Tsuchiya KD Rojas A TGF-beta receptor inactivation and mutant Kras induce intestinal neoplasms in mice via a beta-catenin-independent pathway. Gastroenterology (2009) 136: 1680–8.e7 10.1053/j.gastro.2009.01.066 19208363 PMC2782436

[43] Lu SL Kawabata M Imamura T Miyazono K Yuasa Y Two divergent signaling pathways for TGF-beta separated by a mutation of its type II receptor gene. Biochem Biophys Res Commun (1999) 259:385–90 10.1006/bbrc.1999.0788 10362519

[44] Chen T Yan W Wells RG Rimm DL McNiff J Leffell D Novel inactivating mutations of transforming growth factor-beta type I receptor gene in head-and-neck cancer metastases. Int J Cancer (2001) 93:653–61 10.1002/ijc.1381 11477574

[45] Bellam N Pasche B TGF-beta signaling alterations and colon cancer. Cancer Treat Res (2010) 155:85–103 10.1007/978-1-4419-6033-7_5 20517689

[46] Van Belle P Rodeck U Nuamah I Halpern AC Elder DE Melanoma-associated expression of transforming growth factor-beta isoforms. Am J Pathol (1996) 148:1887–94 8669474 PMC1861638

[47] Wikström P Stattin P Franck-Lissbrant I Damber JE Bergh A Transforming growth factor beta1 is associated with angiogenesis, metastasis, and poor clinical outcome in prostate cancer. Prostate (1998) 37:19–29 10.1002/(SICI)1097-0045(19980915)37:1<19::AID-PROS4>3.0.CO;2-3 9721065

[48] Berking C Takemoto R Schaider H Showe L Satyamoorthy K Robbins P Transforming growth factor-beta1 increases survival of human melanoma through stroma remodeling. Cancer Res (2001) 61:8306–16 11719464

[49] Vázquez PF Carlini MJ Daroqui MC Colombo L Dalurzo ML Smith DE TGF-beta specifically enhances the metastatic attributes of murine lung adenocarcinoma: implications for human non-small cell lung cancer. Clin Exp Metastasis (2013) 30:993–1007 10.1007/s10585-013-9598-1 23832740

[50] Mamura M Lee W Sullivan TJ Felici A Sowers AL Allison JP CD28 disruption exacerbates inflammation in Tgf-beta1-/- mice: in vivo suppression by CD4+CD25+ regulatory T cells independent of autocrine TGF-beta1. Blood (2004) 103:4594–601 10.1182/blood-2003-08-2897 15016653

[51] Garcia-Lazaro JF Thieringer F Lüth S Czochra P Meyer E Renteria IB Hepatic over-expression of TGF-beta1 promotes LPS-induced inflammatory cytokine secretion by liver cells and endotoxemic shock. Immunol Lett (2005) 101:217–22 10.1016/j.imlet.2005.06.003 16054705

[52] Connolly EC Freimuth J Akhurst RJ Complexities of TGF-β targeted cancer therapy. Int J Biol Sci (2012) 8:964–78 10.7150/ijbs.4564 22811618 PMC3399319

[53] Matthews E Yang T Janulis L Goodwin S Kundu SD Karpus WJ Down regulation of TGF-β1 production restores immunogenicity in prostate cancer cells. Br J Cancer (2000) 83:519–25 10.1054/bjoc.2000.1257 10945501 PMC2374659

[54] Zhang Q Yang X Pins M Javonovic B Kuzel T Kim SJ Adoptive transfer of tumor reactive TGF-β insensitive CD8^+^ T cells: eradication of autologous mouse prostate cancer. Cancer Res (2005) 65:1761–9 10.1158/0008-5472.CAN-04-3169 15753372

